# Ultraviolet Radiation and Melanomagenesis: From Mechanism to Immunotherapy

**DOI:** 10.3389/fonc.2020.00951

**Published:** 2020-07-02

**Authors:** Xiaoying Sun, Na Zhang, Chengqian Yin, Bo Zhu, Xin Li

**Affiliations:** ^1^Department of Dermatology, Yueyang Hospital of Integrated Traditional Chinese and Western Medicine, Shanghai University of Traditional Chinese Medicine, Shanghai, China; ^2^Institute of Dermatology, Shanghai Academy of Traditional Chinese Medicine, Shanghai, China; ^3^Department of Cardiology, Longhua Hospital, Shanghai University of Traditional Chinese Medicine, Shanghai, China; ^4^Department of Dermatology, Cutaneous Biology Research Center, Massachusetts General Hospital and Harvard Medical School, Boston, MA, United States; ^5^Department of Cancer Immunology and Virology, Dana-Farber Cancer Institute, Boston, MA, United States

**Keywords:** ultraviolet radiation, melanomagenesis, melanoma, immunotherapy, cytotoxic T-lymphocyte-associated protein 4, programmed cell death protein 1

## Abstract

Melanoma is the deadliest form of skin cancer, and nearly 90% of melanomas are believed to be caused by ultraviolet radiation (UVR), mainly from sunlight. UVR induces DNA damage, forming products such as cyclobutane pyrimidine dimers (CPD) and 6-4-pyrimidone photoproducts (6-4PP) in a wavelength-dependent manner and causes oxidative DNA damage. These DNA lesions lead to DNA mutations and contribute to the formation of melanoma. In this review, we discuss the protective role of melanocytes against UV-induced DNA damage and how genetic variations, including those in p53 and melanocortin-1 receptor (MC1R), or epigenetic histone modifications in melanocytes result in a tendency toward melanoma. We also provide a summary of prevention and treatment strategies against melanoma, including the most recent immunotherapies. Collectively, this work contributes to the understanding of the molecular pathogenesis of UV-induced melanoma.

## UV and Melanomagenesis

**Figure 1:**
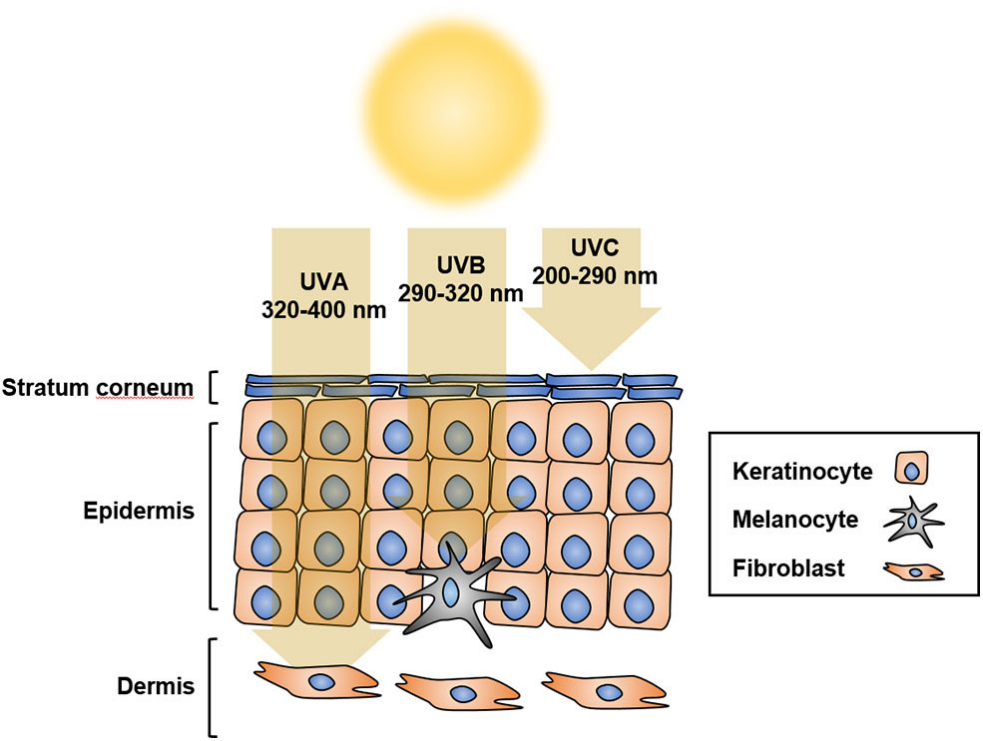
UV penetration into the skin. UV radiation has shorter wavelength than visible light, which makes it invisible to the naked eye. Based on different wavelengths, UV radiation is classified as UVA at 320 to 400 nm, UVB at 290 to 320 nm, or UVC at 200 to 290 nm. UVA penetrates the skin more deeply than UVB and UVC, but does not damage the epidermis considerably. UVB causes sunburn and damages the epidermis, thus playing a central role in the development of skin cancer. Most UVC does not reach the earth because of its short wavelength, and is absorbed by the ozone layer.

Solar ultraviolet radiation (UVR) is considered to be the main etiological factor for skin cancer, including melanoma. UVR comprises ultraviolet C (UVC; 200–290 nm), ultraviolet B (UVB; 290–320 nm), and ultraviolet A (UVA; 320–400 nm) (1). UVR is the major environmental risk factor for melanoma development (2). UVB causes sunburn and damages the epidermis, thus playing a central role in the development of skin cancer, whereas UVA penetrates the skin deeper than UVB or UVC, but does not damage the epidermis considerably; UVC, however, penetrates the deeper layers of the skin limitedly (3) (Figure 1). The direct consequence of solar UV is generation of DNA photoproducts, mainly cyclobutane pyrimidine dimers (CPD) and pyrimidine 6–4-pyrimidone photoproducts (6-4PP). In addition, UV-induced reactive oxygen species (ROS) indirectly cause oxidative DNA damage (4–6). UV-induced damage to cells and tissues includes DNA mutations and altered DNA integrity, transcription profile, and protein modification, which result in the dysregulation of multiple oncogenes and tumor suppressor genes (7, 8).

**Figure 2:**
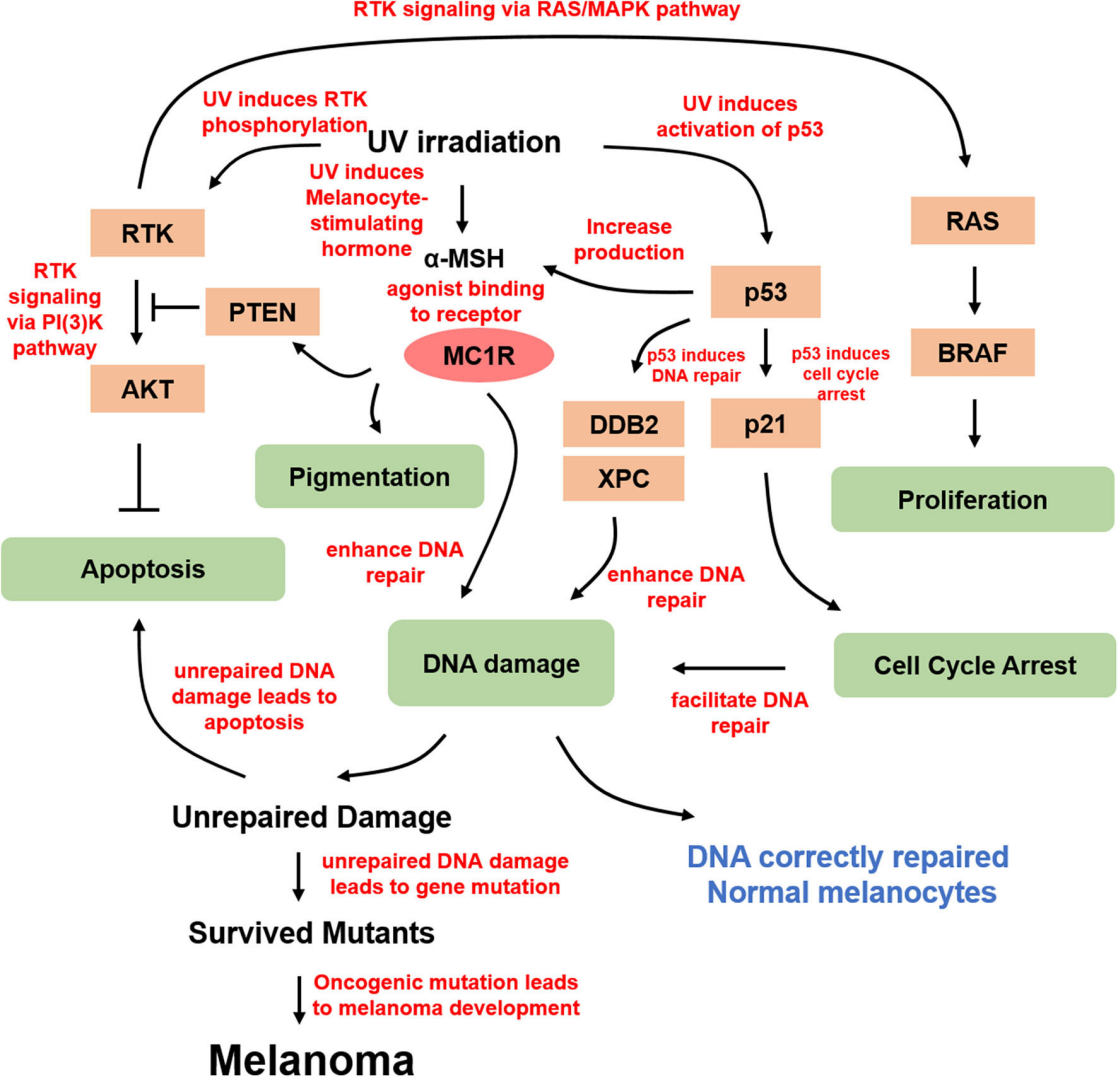
Molecular mechanism of UV-derived melanomagenesis. Direct UV irradiation results in DNA damage, typically in the form of CPD and 6-4PP, which are generated from UVB. α-MSH is the most important melanocortin stimulated by UV irradiation, thereby activating MC1R for melanogenesis and DNA damage repair. In addition, α-MSH/MC1R stabilizes PTEN upon UVB stimulation. UV irradiation also activates p53 and its downstream targets, including p21 in proliferating melanocytes arresting cell cycle at G1 or G2 prior to repair, and several genes from the xeroderma pigmentosum (XP) gene family involving XPC and damage-specific DNA binding protein 2 (DDB2), which is a product of XPE, stimulating nucleotide excision repair for efficient DNA damage repair. DNA damage-induced mutant melanocytes may constitute a pool of cells in which melanoma develops eventually. Moreover, UV irradiation directly activates RTKs, in turn activating several essential pathways, including anti-apoptotic signaling through AKT and proliferation signaling through MAPK.

There are three types of skin cancers: basal cell carcinoma, squamous carcinoma, and melanoma. Basal cell carcinoma and squamous carcinoma, also known as non-melanoma skin cancer, account for nearly 98% of all skin cancer cases in the United States (9). Although melanoma accounts for the least number of cases of all skin cancers, because of its high metastatic potential and resistance to chemotherapy and radiation therapy, melanoma is responsible for the majority of skin cancer-related deaths (10). Compared to the incidence of non-melanoma skin cancers, the incidence of melanoma is increasing. In 2020, ~100,350 new cases of melanoma and 6,850 deaths are expected (11). Similar to other types of solid tumors, melanoma shows frequent alterations in MAPK and PI3K/PTEN signaling pathways, especially with 40–60% of cultured primary melanoma cells bearing activating BRAF mutations (12–20) (Figure 2). Constitutive activation of BRAF kinase through mutations is the most common way for melanoma to activate the MAPK pathway (20). The most common oncogenic mutation for BRAF kinase is the substitution of amino acid valine for glutamic acid at position 600 (V600E) accounting for nearly 90% of the BRAF mutations. In addition, there are other less common V600 mutations such as V600K, V600R, V600M, and V600D, as well as some non-V600 mutations such as K601E and D594N (21). Targeting the dominant activating mutation BRAF V600E, which activates the MAPK pathway leading to uncontrolled proliferation, with type I RAF inhibitor vemurafenib showed promising clinical benefits (22). However, specific inhibitors of PI3K/AKT were developed and assessed in clinical trials, providing translational potentials (23). However, after the initial tumor response, melanoma cells acquired resistance to the inhibitors, indicating secondary alterations or mutations (24). Intriguingly, mutated BRAF cooperates with alterations in PTEN/AKT to promote melanoma progression (25). Therefore, targeting both signaling pathways simultaneously was proposed for melanoma treatment (26).

The UV-induced DNA damage response pathway is modulated by the tumor suppressor p53, whose functional deletion drives UV-mediated mutagenesis in melanoma, squamous cell carcinoma, basal cell carcinoma, and actinic keratosis (27). Tumor suppressor p53 and its related gene products play important roles under different cellular conditions. Accumulation of p53, stimulated by UVR, is required for inducing cell cycle arrest, DNA repair, and apoptosis (28, 29). UV-irradiated p53 knockout mice show a higher incidence of skin tumor development (30). G1/G2 arrest is required for repairing UV-induced DNA damage (31, 32). P53 upregulation directly induces the expression of cyclin-dependent kinase (CDK) inhibitor p21, which mediates UV-induced DNA damage at G1 cell cycle arrest. In addition, the accumulation of p53 also activates several genes from the xeroderma pigmentosum (XP) gene family, including XPC and damage-specific DNA binding protein 2 (DDB2), which is a product of XPE, to stimulate nucleotide excision repair (NER) for efficient DNA damage repair (33). TP53/Trp53 was also shown to cooperate with BRAF V600E to induce melanoma in the presence of UVR (34). Moreover, p53 was found to potently stimulate the proopiomelanocortin (POMC) promoter and induce the generation of melanocortin peptides in response to UV, and research based on a transgenic knockout mouse model demonstrated that p53 loss results in the absence of the UV-tanning response (35). Therefore, p53 functions as a sensor and effector for UV-induced pigmentation, providing insight into the treatment of pathologic hyperpigmentation due to tanning response.

Melanocytes play an important photoprotective role in response to UV exposure via melanin synthesis. Melanocyte-produced melanin protects nuclear DNA from UV irradiation and reduces the generation of DNA damage (36). Melanin within melanosomes of melanocytes is transferred to keratinocytes where they cause tanning, which is a hallmark of UV exposure, mediated by multiple paracrine factors synthesized by keratinocytes (37–39). There are two major forms of melanin generated by melanocytes, eumelanin and pheomelanin. Eumelanin is dark brown or black, whereas pheomelanin is red or orange. It is known that pheomelanin is associated with type I/II skin, freckles, red hair, and an inability to tan (40). The diversity of skin pigmentation phenotypes among individuals from distinct ethnic groups is mainly due to the difference in the content of eumelanin and pheomelanin in melanocytes (41, 42). Studies show that skin cancer, including melanoma, is inversely related to skin pigmentation, with lower skin cancer incidence in individuals with dark skin and higher incidence in individuals with fair skin (43–47). Consistent with these findings, increased eumelanin content was observed to reduce the generation of DNA photoproducts following UV exposure (48).

UVR induces the synthesis and release of melanocortin peptides, including α-melanocyte-stimulating hormone (α-MSH), in both melanocytes and keratinocytes to activate melanocortin 1 receptor (MC1R) signaling (49, 50). MC1R is a highly conserved G protein-coupled receptor expressed on the surface of melanocytes (51). MC1R transduces extracellular signals mediated by melanocortins to downstream effectors including microphthalmia-associated transcription factor (MITF), to regulate skin pigmentation and control cell proliferation and apoptosis (52). In melanocytes, the activation of the MC1R signaling pathway stimulates tyrosinase (TYR) activity, which is the rate-limiting enzyme of melanin production (53). Therefore, MC1R plays a critical role in protecting the skin from UVR.

MC1R is identified as an important factor in preventing melanoma formation. The activation of MC1R by UVR induces adenylate cyclase, cAMP production, protein kinase A activation, melanin synthesis, and downstream UV protective genes such as MITF, tyrosinase, and TRP1 (54–56). Studies show that UVR can induce MSH expression, but fails to stimulate pigmentation in the absence of functional MC1R in red/blonde-haired MC1R transgenic mice. A cyclic AMP agonist forskolin was applied topically, and the resulting chemically induced pigmentation was protective against UV-induced cutaneous DNA damage and tumorigenesis (50). A systematic study on the contribution of MC1R to somatic mutations in sporadic melanoma found that individuals with germline disruptive variants in MC1R have a significantly higher somatic mutational load. This study is the first to report the role of germline MC1R variants in influencing the somatic mutational landscape of melanoma from human data (57). The MC1R mechanistic study showed that the MC1R–PTEN axis serves as a central regulator in response to UVB exposure in melanocytes, which reveals the molecular basis underlying the association between MC1R variants and melanomagenesis. The study indicated that MC1R variants are defective in association with PTEN following UV exposure, consequently failing to suppress the PI3K/AKT signaling pathway, and the MC1R deficiency-induced elevation in PI3K/AKT signaling drives oncogenic transformation in melanoma (58).

Individuals carrying MC1R variants, especially those associated with red hair color, fair skin, and poor tanning ability [red hair color (RHC) variants], have a higher risk of developing melanoma (59). However, how MC1R activity is modulated by UVR and why individuals with red hair are more prone to develop melanoma remain unclear. A recently published study used mouse models to demonstrate a potential MC1R-targeted intervention strategy to rescue loss-of-function MC1R for therapeutic benefits. Studies show that MC1R palmitoylation, primarily mediated by the protein acyltransferase ZDHHC13, is essential for activating MC1R signaling. The activated MC1R signaling increased pigmentation, UVB-induced G1 cell cycle arrest, and control of senescence both *in vitro* and *in vivo* (60, 61). Using transgenic mice expressing MC1R RHC variants, studies showed that pharmacological activation of palmitoylation rescues the functional defects of MC1R RHC variants and prevents melanomagenesis. The results highlight a central role for MC1R palmitoylation in pigmentation and protection against melanoma (61).

## UV and Immunosuppression

Besides inducing melanomagenesis, UVR can suppress immunity in several ways, including inhibition of antigen presentation, the release of immunosuppressive cytokines, and apoptosis of immune cells. UVR-suppressed immunity contributes to the clearance of tumor cells.

Cutaneous immunity depends on the proper functioning of epidermal Langerhans cells (LCs), which are the main antigen-presenting cells (APCs) in the skin. UVR directly damages LCs with decreased cell numbers and inhibition of antigen-presenting function (62). UV-irradiated LCs lose the ability to stimulate T-helper 1 (Th1) cells in response to foreign antigens, and preferentially activate Th2 cells to promote suppressor T cell function (63). Spleen cells from mice treated with UVR fail to present antigen to Th1 cells. However, this failure could be reversed by injecting anti-IL-10 antibodies into these mice. In this rescue experiment, the antigen-presenting ability of LCs was restored and the LCs effectively activated the Th1 cells. Moreover, the administration of anti-IL-10 antibodies could significantly inhibit UVR-induced antigen presentation of LCs to Th2 cells. The repression may be mediated by suppressive cytokines, such as IL-4 and IL-10, released by the induced T suppressor cells (64, 65).

TNF-α is another UVR-modulated immunosuppressive cytokine (66, 67). Mice treated with anti-TNF-α antibody showed a significant decrease in LCs (68). The pro-inflammatory cytokine IL-12 played an important role in the activation of Th1 cells and blockade of Th2 cells. UVR exposure significantly reduced IL-12 expression, resulting in the suppression of Th1 and de-repression of Th2 cells (69). Another study reported similar results where treatment of mice with IL-12 strongly inhibited UV-induced suppressor T cells *in vivo* (70).

Skin cancer cells are highly antigenic and could be forcefully rejected by mice. However, UVR-treated mice fail to reject these cancer cells, suggesting that UV-induced immunosuppression promotes tumor growth and progression *in vivo* (71). UV-induced immunosuppression results from increased T suppressor cells or T regulatory cells, which enhances immune tolerance to tumor antigens (72). This study also suggests that regulatory T cells could be targeted as a vital effector to inhibit UVB-induced immunosuppression, thus enhancing anti-tumor immunity. Therefore, UVB-induced skin cancer is not only caused by UV-induced DNA lesions but also fueled by the generation and maintenance of an immunosuppressed tumor microenvironment (73).

## Immunotherapies in Melanoma

The immune system plays a critical role in clearing neoplastic cells. Evading the immune system is crucial for tumor cell survival and proliferation. Treatment of the most deadly form of skin cancer, metastatic melanoma (74), has advanced tremendously in the last decade, especially targeted therapy and immunotherapy. Currently, several types of immunotherapies are being studied to treat melanoma (Table 1).

**Table 1:** Key findings of immunotherapies on melanoma.

**Types of treatment**	**Clinical trial**	**Phase**	**Status**	**Population**	**Treatment arms**	**Number of patients**	**Primary outcome**		**95% CI**	**HR**	***P*-value**
Cytokines	(74)	II	Completed	Disseminated malignant melanoma	IFN-α, 12U × 10^6^/m^2^ or 50U × 10^6^/m^2^, Q3W	96	ORR (%)	22	-	-	-
	(75)	/	Completed	Progressive metastatic melanoma	IL-2, 22 or 33 or 36 or 44 μg/kg, Q8H	270	ORR (%)	16	12 to 21	-	-
	(76) (NCT00287131)	II	Unknown	Stage IV melanoma	Infusion of TIL + IL-2 720,000 U/kg Q8H	20	ORR (%)	50	-	-	-
	(77)	II	Unknown	Metastatic melanoma	Chemotherapy + infusion of TIL + IL-2 720,000 U/kg Q8H	43	ORR (%)	49	-	-	-
					2 Gy of total-body irradiation + infusion of TIL + IL-2 720,000 U/kg Q8H	25	ORR (%)	52	-	-	-
					12 Gy of total-body irradiation + infusion of TIL + IL-2 720,000 U/kg Q8H	25	ORR (%)	72	-	-	-
Inhibitors of immune checkpoints	(78) (NCT00324155)	III	Completed	Untreated unresectable stage III or IV melanoma	Dacarbazine, 850 mg/m^2^, Q3W + ipilimumab, 10 mg/kg, Q3W	250	OS (mo)	11.2	9.4 to 13.6	0.72	*P* < 0.001
					Dacarbazine, 850 mg/m^2^, Q3W + placebo, 10 mg/kg, Q3W	252	OS (mo)	9.1	7.8 to 10.5	-	-
	(79) (NCT00094653)	III	Completed	Previously treated, unresectable Stage III or IV melanoma	gp100 + placebo, 3 mg/kg, Q3W	136	OS (mo)	6.4	5.5 to 8.7	-	-
					gp100 + ipilimumab, 3 mg/kg, Q3W	403	OS (mo)	10	8.5 to 11.5	0.68 (vs. gp100)	*P* < 0.001
					Placebo + ipilimumab, 3 mg/kg, Q3W	137	OS (mo)	10.1	8.0 to 13.8	0.66 (vs. gp100)	*P* = 0.003
	(80) (NCT00257205)	III	Completed	Stage IIIc or IV melanoma	Tremelimumab, 15 mg/kg, Q90D	328	OS (mo)	12.6	10.8 to 14.3	0.88	*P* = 0.127
					Investigator-choice chemotherapy	327	OS (mo)	10.7	9.36 to 11.96	-	-
	(81) (NCT01295827, KEYNOTE-001)	I	Completed	Previously treated, progressive, measurable, unresectable melanoma	Pembrolizumab, 2 mg/kg, Q3W	89	ORR (%)	27	18 to 37	-	*P* = 0.46
					Pembrolizumab, 10 mg/kg, Q3W	84	ORR (%)	32	22 to 43	-	-
	(82) (NCT01704287, KEYNOTE-002)	II	Completed	Previously treated, progressive, advanced melanoma	Pembrolizumab, 2 mg/kg, Q3W	180	PFS (mo)	4.2	3.1 to 6.2	0.57 (vs. chemo)	*P* < 0.0001
					Pembrolizumab, 10 mg/kg, Q3W	181	PFS (mo)	5.6	4.2 to 7.7	0.50 (vs. chemo)	*P* < 0.0001
					Investigator-choice chemotherapy	179	PFS (mo)	2.6	2.5 to 2.8		
	(83) (NCT01866319, KEYNOTE-006)	III	Completed	Previously treated, unresectable stage III or IV melanoma	Pembrolizumab, 10 mg/kg Q2W	279	PFS (mo)	5.5	3.4 to 6.9	0.58 (vs. ipi)	*P* < 0.001
					Pembrolizumab, 10 mg/kg Q3W	277	PFS (mo)	4.1	2.9 to 6.9	0.58 (vs. ipi)	*P* < 0.001
					Ipilimumab, 3 mg/kg, Q3W	278	PFS (mo)	2.8	2.8 to 2.9	-	-
	(84) (NCT00730639)	I	Active, not recruiting	Advanced melanoma	Nivolumab, 1, 3, or 10 mg/kg Q2W	107	OS (mo)	16.8	12.5 to 31.6	-	-
	(85) (NCT01721772, CheckMate 066)	III	Active, not recruiting	Metastatic melanoma without a BRAF mutation	Nivolumab, 3 mg/kg, Q2W	210	1-year OS rate (%)	72.9	65.5 to 78.9	0.42	*P* < 0.001
					Dacarbazine, 1000 mg/m^2^, Q3W	208	1-year OS rate (%)	42.1	33.0 to 50.9	-	-
Combination therapy with anti-CTLA4 and anti-PD-1	(86, 87) (NCT01927419)	II	Active, not recruiting	Unresectable, previously untreated, stage III, or IV melanoma	Ipilimumab, 3 mg/kg, Q3W + nivolumab, 1 mg/kg, Q3W	94	ORR (%)	61.1	48.9 to 72.4	-	*P* < 0.001
							24-month OS rate (%)	63.8	53.3 to 72.6	0.74	*P* = 0.26
					Ipilimumab, 3 mg/kg, Q3W + placebo, 1 mg/kg, Q3W	46	ORR (%)	10.8	3.0 to 25.4	-	-
							24-month OS rate (%)	53.6	38.1 to 66.8	-	-
	(88–90) (NCT01844505, CheckMate 067)	III	Active, not recruiting	Unresectable, previously-untreated, stage III, or IV metastatic melanoma	Nivolumab 3 mg/kg, Q2W + placebo for ipilimumab, 3 mg/kg, Q3W	316	PFS (mo)	6.9	4.3 to 9.5	0.57 (vs. ipi)	*P* < 0.00001
							36-month OS (mo)	37.6	29.1 to NR	0.65 (vs. ipi)	*P* < 0.001
							60-month OS (mo)	36.9	28.2 to 58.7	0.63 (vs. ipi)	*P* < 0.001
					Nivolumab 3 mg/kg, Q2W + ipilimumab, 3 mg/kg, Q3W	314	PFS (mo)	11.5	8.9 to 16.7	0.42 (vs. ipi)	*P* < 0.00001
							36-month OS (mo)	NR	38.2 to NR	0.55 (vs. ipi)	*P* < 0.001
							60-month OS (mo)	NR	38.2 to NR	0.52 (vs. ipi)	*P* < 0.001
					Placebo for nivolumab 3 mg/kg, Q2W + ipilimumab, 3 mg/kg, Q3W	315	PFS (mo)	2.9	2.8 to 3.4	-	-
							36-month OS (mo)	19.9	16.9 to 24.6	-	-
							60-month OS (mo)	19.9	16.9 to 24.6	-	-
	(91) (NCT02977052)	II	Recruiting	Resectable, stage III metastatic melanoma	Ipilimumab, 3 mg/kg, Q3W + nivolumab, 1 mg/kg, Q3W	30	ORR (%)	63	44 to 80	-	-
					Ipilimumab, 1 mg/kg, Q3W + nivolumab, 3 mg/kg, Q3W	30	ORR (%)	57	37 to 75	-	-
					Ipilimumab, 3 mg/kg, Q3W + nivolumab, 3 mg/kg, Q2W	26	ORR (%)	35	17 to 56	-	-
	(92) (NCT03165422)	-	Unknown	Unresectable melanoma	Nivolumab to ipilimumab	61	ORR (%)	4.9	-	-	-
					Ipilimumab to nivolumab	7	ORR (%)	20	11.4 to 31.3	-	-
	(93)	-	Unknown	Treatment naïve, unresectable stage IIIC/IV melanom	Ipilimumab, 3 mg/kg, Q3W + nivolumab, 3 mg/kg, Q2W	60	PFS (mo)	11	6.0 to NR	-	-
				Refractory to first-line BRAF therapy, unresectable stage IIIC/IV melanoma		33	PFS (mo)	2	1.4 to 4.6	-	-
				Prior PD-1 inhibitor therapy, unresectable stage IIIC/IV melanoma		57	PFS (mo)	4	2.8 to NR	-	-
Combination therapy based on anti-CTLA4 or anti-PD-1	(94) (NCT01968109)	II	Recruiting	Previously anti-PD-1/PD-L1-treated, progressive melanoma	BMS-986016 + nivolumab	43	ORR (%)	16	-	-	-
	(95) (NCT02752074, KEYNOTE-252)	III	Completed	Unresectable stage III or stage IV melanoma	Pembrolizumab 200 mg Q3W + epacadostat 100 mg BID	354	PFS (mo)	4.7	2.9 to 6.8	1	*P* = 0.52
					Pembrolizumab 200 mg Q3W + placebo 100 mg BID	352	PFS (mo)	4.9	2.9 to 6.8	-	-
	(96) (NCT01740297)	II	Active, not recruiting	Unresectable stages IIIB to IV melanoma	Talimogene Laherparepvec ≤ 4 ml × 10^8^ plaque-forming U/ml + ipilimumab 3 mg/kg, Q3W	98	ORR (%)	39	-	-	*P* = 0.002
					Ipilimumab 3 mg/kg, Q3W	100	ORR (%)	18	-	-	-
	(97)	Ib	Unknown	Advanced melanoma	Oncolytic viral injection, 4 ml × 10^8^ pfu/ml, Q2W + pembrolizumab, 200 mg, Q3W	21	ORR (%)	62	38 to 82	-	-
	(98)	-	Unknown	Bone metastatic, stage IV melanoma	Nivolumab + ipilimumab + denosumab	13	ORR (%)	54	-	-	-
					PD-1-inhibitor + denosumab	16	ORR (%)	50	-	-	-

### Cytokines (Interferon-α and Interleukin-2)

One common method used to boost the immune system is by treatment with cytokines. For example, both interferon-α (IFN-α) and interleukin-2 (IL-2) are administered to melanoma patients. A clinical study found that IFN-α shrinks advanced melanomas with tumor response rates of about 22% and median times to progression and survival of 1.5 and 5 months (99). Intriguingly, tumor burden is found to affect the response. A higher potential of response is observed among patients who have a lower tumor burden. The different immune responses in patients with advanced tumors from those with lower tumor burden indicates different benefits and risk for recurrence and death in patients treated with IFN-α. This hypothesis opened the door for the initial evaluation of adjuvants with IFN-α in melanoma (100). Studies report that IFN-α has a significant effect on activating STAT signaling. The treatment with IFN-α was involved in the significant promotion of tumor-infiltrating dendritic cells (DCs) and T cells (101). In addition, the type I IFN signature is associated with an effector immune response to melanoma in both mouse models and human melanoma patients (75).

Metastatic melanoma trials showed that IL-2 treatment shrinks advanced melanomas with an objective response rate of 16%, with a median duration of response of 8.9 months (76). Intriguingly, a combination of IL-2 treatment with an infusion of *ex vivo* expanded TIL after chemotherapy-induced lymphodepletion or total body radiotherapy increased the response rate to about 50–72% in metastatic melanoma patients (77, 102). IL-2 is produced by activated CD4^+^ T, CD8^+^ T cells, NK cells, and DCs (103). IL-2 boosts the effector lymphocyte immune response and plays an essential role for the IL-2 receptor in immunosuppressive regulatory T cells (104).

### Inhibitors of Immune Checkpoints

To enhance anti-tumor immunity, the most successful immunotherapeutic strategy is the use of monoclonal antibodies to block immunoregulatory suppression in the tumor microenvironment.

**Figure 3:**
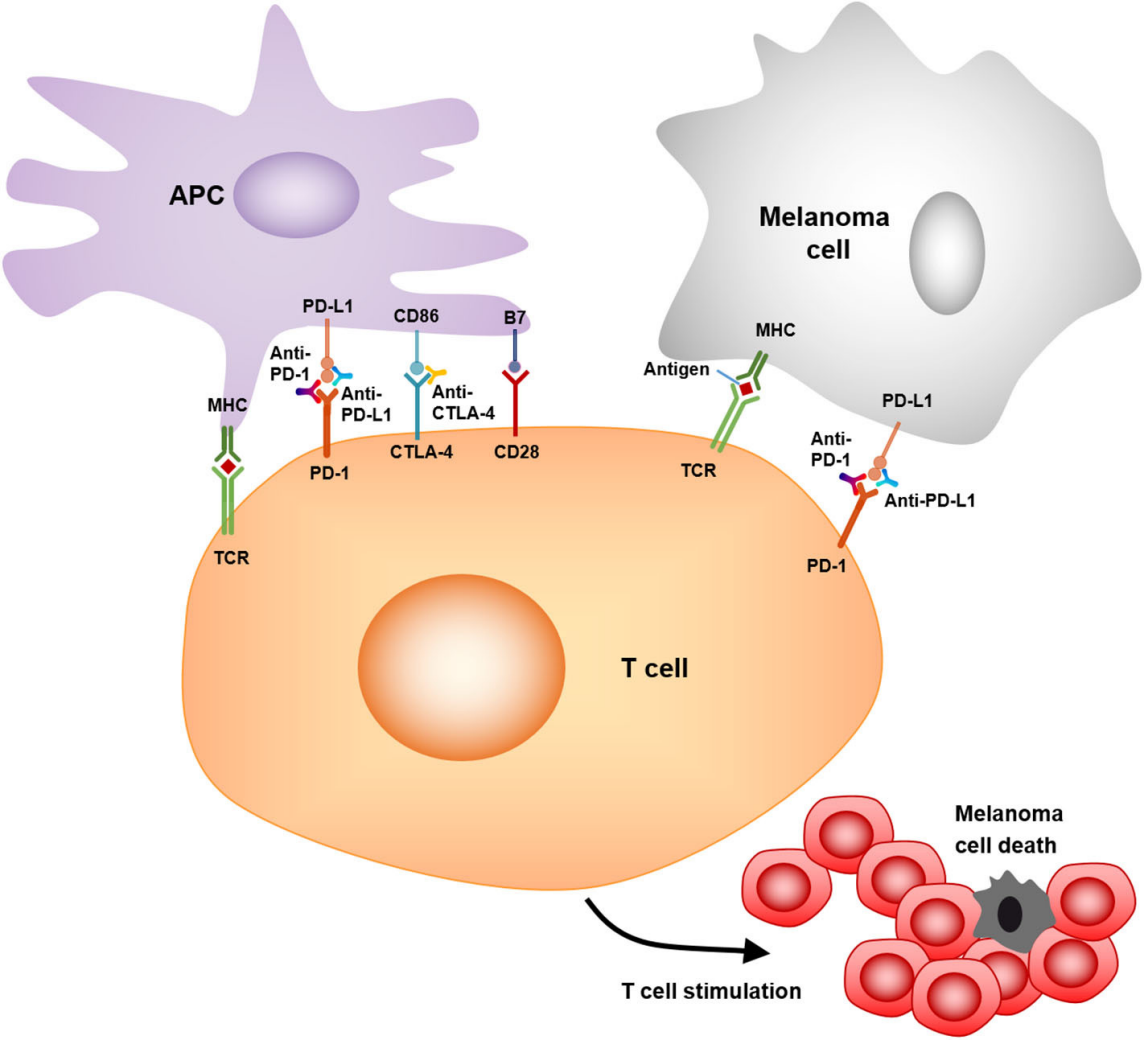
Mechanism of action of anti-PD-1, anti-PD-L1, and anti-CTLA4. PD-1/PD-L1, CTLA-4/CD86 binding inhibits T cell killing of melanoma cells. Blocking PD-1, PD-L1, or CTLA-4 allows T cell killing, APC–T cell interaction, and T cell stimulation (i.e., cytokine secretion, lysis, proliferation, and migration to melanoma) in a melanoma microenvironment.

As a member of the CD28:B7 immunoglobulin superfamily, cytotoxic T-lymphocyte-associated protein 4 (CTLA-4) is normally expressed at low levels on naïve effector T cells and regulatory T cells (Tregs), but is strongly induced after activation. The upregulated CTLA-4 competes with CD28 for binding with B7, which suppresses T cell receptor signaling to inhibit immunity (105). The anti-CTLA-4 monoclonal antibodies, ipilimumab, and tremelimumab were used in clinical trials to blockade the CTLA-4 signaling, which causes T cell proliferation, activation, and infiltration. Amplification of T cell-mediated immunity and enhanced anti-tumor immune response in the tumor microenvironment were observed (78) (Figure 3). The clinical data show that ipilimumab is a promising drug in treating cancer. The overall survival (OS) was extended in the treated patients (11.2 vs. 9.1 months; HR 0.72; *p* < 0.001) (79). Moreover, the combination of ipilimumab with the gp100 peptide, a promising anti-tumor vaccine, has improved gp100 peptide benefits with the OS extending from 6.4 to 10.0 months (80). In addition, another ipilimumab clinical trial showed that the treatment improved median OS to 11.4 months (95% CI, 10.7 to 12.1 months), with a plateau at 21% in the survival curve beginning around 3 years. Tremelimumab also shows promising clinical activity in advanced melanoma when tested in a phase III clinical trial (A3671009). The OS is 12.6 months with a 1-year survival rate of more than 50%, compared to 10.7 months for chemotherapy (106).

Programmed cell death protein 1 (PD-1) and programmed cell death-ligand 1 (PD-L1) are also important immunotherapeutic targets for melanoma. PD-1/PD-L1 signaling has been shown to inhibit tumor effector CD8+ T cells. PD-L1 expression is observed in many tumors, including melanoma (107). High expression of PD-L1 on melanoma cells correlates with poor prognosis and low survival rate (108). The PD-1/PD-L1-induced immunosuppression in the microenvironment has resulted in tumor resistance to cytotoxic T cell response (81, 107). Clinical trials showed that treatment with pembrolizumab, an anti-PD-1 antibody, has promising benefits for cancer patients. The overall response rate was 18–43%, and these responses were robust, with OS of 69% at 1 year (82). Another phase II clinical trial with KEYNOTE-002 showed a higher 6-month progression-free survival compared to chemotherapy (38 vs. 16%) (83). Moreover, a phase III trial with ipilimumab and pembrolizumab showed that the overall response rate (ORR) was 33% (pembrolizumab) vs. 12% (ipilimumab). The OS rates for 1 year were 68–74% for pembrolizumab vs. 58% for ipilimumab. Therefore, pembrolizumab was superior to ipilimumab in this study (84).

As a fully human anti-PD-1 monoclonal antibody, nivolumab showed an OS of 17.3 to 20.3 months (85). Nivolumab became the second monoclonal antibody against PD-1 receptor to be approved by the FDA for the treatment of patients with unresectable or metastatic melanoma and disease progression following ipilimumab and a BRAF inhibitor (if BRAF V600 mutation-positive). The study of phase III trials on patients with metastatic melanoma showed an overall response rate of 32% for nivolumab treatment vs. 11% for chemotherapy (109).

## Combination Immunotherapy in Melanoma

### Combination Therapy With Anti-CTLA4 and Anti-PD-1

The emergence of combination immunotherapy has greatly improved the poor survival outcome of patients with advanced melanoma in the past with a median OS time of about 8 months, and a 5-year survival rate of about 10% from patients diagnosed with metastatic diseases (86). The results of a phase II trial (CheckMate 069) showed that the combination of nivolumab and ipilimumab demonstrated a statistically significant improvement in objective response rate and longer progression-free survival in treatment-naïve patients with BRAF wild-type melanoma compared with ipilimumab alone (87). The two-year OS rate was 63.8% (95% CI, 53.3–72.6) in the combination group and 53.6% (95% CI, 38.1–66.8) in the ipilimumab alone group (88). Recently, a phase III trial of treatment-naïve patients with advanced melanoma (CheckMate 067) showed that nivolumab in combination with ipilimumab leads to longer progression-free survival and a higher objective response rate than ipilimumab alone (89). The results of the 3-year and 5-year overall data reports for this trial were observed as follows. During the follow-up period of at least 36 months, the median OS time of the nivolumab plus ipilimumab group was not reached, and the median OS time of the nivolumab group was 37.6 months, while that of the ipilimumab group was 19.9 months. The overall 3-year survival rate was 58% in the nivolumab plus ipilimumab group, 52% in the nivolumab group, and 34% in the ipilimumab group, and the security data were not changed from the original report (90). During a follow-up of at least 60 months, the median OS time was more than 60.0 months in the nivolumab plus ipilimumab group, 36.9 months in the nivolumab group, and 19.9 months in the ipilimumab group. Compared with 26% in the ipilimumab group, the 5-year OS rate was 52% in the nivolumab plus ipilimumab group and 44% in the nivolumab group. Patients who received the nivolumab regimen had no significant loss of quality of life such as new late toxic effects (110).

Although both combination therapy and nivolumab monotherapy were more effective than ipilimumab, the frequency of grade 3/4 adverse events (AEs) induced by combination therapy with a dose of ipilimumab (3 mg/kg) plus nivolumab (1 mg/kg) once every 3 weeks was higher than that of nivolumab monotherapy. However, the difference in grade 3/4 AEs between the two groups did not translate into the reported difference in the clinical significance of health-related quality of life (HRQL), further supporting the clinical benefits of nivolumab monotherapy and the combination of nivolumab and ipilimumab in the treatment of advanced melanoma (91). For more extensive clinical application, the early observations of the OpACIN-NEO trial confirmed that two cycles of ipilimumab (1 mg/kg) plus nivolumab (3 mg/kg) once every 3 weeks intravenously could be used as a less toxic but equally effective dose plan for ipilimumab plus nivolumab (92). In addition to combination therapy, sequential therapy is also common in clinical applications. Another retrospective study describing the treatment patterns of nivolumab and ipilimumab observed that switching from nivolumab to ipilimumab was common in Japanese melanoma patients, and the independent factors of high neutrophil-to-lymphocyte ratio and high C-reactive protein before nivolumab treatment could predict the poor prognosis of progression-free survival (111).

To supplement the traditional survival endpoint, it is necessary to fully capture the result measurement method of immuno-oncology drug properties. The concept of treatment-free survival (TFS) is proposed, which can characterize the anti-tumor activity and refer to the period from the discontinuation of immune checkpoint inhibitor therapy caused by adverse events to the subsequent systemic treatment or death (112). TFS analysis of CheckMate 067 and 069 showed that patients with advanced melanoma treated with nivolumab plus ipilimumab had longer TFS and less toxicity than those who received nivolumab or ipilimumab (93).

Although the combination of ipilimumab and nivolumab is a highly effective systematic therapy for metastatic melanoma, patients with BRAF mutations who failed in previous target therapy of BRAF/MEK inhibitors showed less response, and the median progression-free survival was only 2.0 months (95% CI, 1.4–4.6) (113). The study of ipilimumab and nivolumab in patients with metastatic melanoma showed that soft tissue and lung metastasis had the highest lesional response rate (79 and 77%, respectively), while liver metastasis had the lowest (46%), suggesting that specific disease sites may have unique response patterns and drug resistance mechanisms, and personalized treatment should be allowed (94).

### Combination Therapy Based on Anti-CTLA4 or Anti-PD-1

The early clinical trials of the new immunotherapy combination also proved that blocking PD-1 combined with other new immunoregulatory targets is safe and effective. Nivolumab combined with anti-lymphocyte activating gene 3 (LAG-3) antibody had an objective response in 31 patients with prior disease progression, with an objective effective rate of 16% and a disease control rate of 45% (95). The I/II phase trial of pembrolizumab and indoleamine 2-dioxygenase 1 (IDO1) inhibitor epacadostat has also shown preliminary efficacy, but the combined randomized III phase trial did not show any additional benefits compared with pembrolizumab alone (114). At present, several clinical trials (NCT01968109, NCT03743766, NCT02676869, and NCT03470922) are underway to combine PD-1 blocking with LAG-3 blocking or IDO1 blocking. In addition, phase I/II trials (NCT02817633, NCT02608268, NCT03099109, and NCT03066648) are being conducted to analyze the efficacy and safety of dual anti-T cell immunoglobulin and mucin 3 (MBG453) and anti-PD-1 (PDR001) effects.

The effectiveness of BRAF/MEK inhibitors has been explored to combine it with immunotherapy. However, despite the promising combination of vemurafenib (BRAF inhibitor) and ipilimumab, the first phase I study was discontinued early because of unexpectedly high hepatotoxicity (96). Other combinations that can improve the therapeutic effect are being explored. In a phase II study, the objective response rate of talimogene laherparepvec combined with ipilimumab was significantly higher, without additional safety issues (97). The combination of pembrolizumab with talimogene laherparepvec has also demonstrated safety and effectiveness in an early phase study (115), and a randomized phase III trial is underway (NCT02263508). Targeting the Wnt/β-catenin signaling pathway should be a high priority for combination therapy to improve the efficacy of anti-CTLA4 or anti-PD-1 agents (98). In addition, the combination of PD-1 inhibitor and denosumab that inhibits receptor activator of nuclear factor kappa-B ligand (RANKL), which promotes osteoclast formation and has an immunological effect, showed good therapeutic signals in patients with bone metastasis from melanoma (116). Although most melanoma immunotherapy evaluation trials exclude brain metastasis patients, it is commendable that local therapy (stereotactic radiosurgery, surgery, or laser interstitial thermal therapy) continues to play an important role in the treatment of melanoma patients with brain metastases receiving immunotherapy (117), and combination of radiotherapy and immunotherapy can even improve the survival rate (118).

## Conclusions and Prospects

Exposure to the sun or UVR and the sensitivity of an individual's skin are risk factors for melanoma. In a skin protection behavior study, melanoma survivors were encouraged to use sunscreen and seek shade more frequently. The study stressed that melanoma survivors should always use sunscreen and seek shade to protect their health (119).

It remains significantly important to understand UV-associated skin pathology and identify therapeutic strategies to bypass resistance and increase the proportion of response in order to advance melanoma research. A few previously incurable metastatic melanomas now have potential cures. Complementary insights from melanoma and immunology studies are essential for the development of novel therapeutic strategies and combination rationales for melanoma treatment (120).

Immunotherapy, especially against CTLA-4 and PD-1/PD-L1 signaling to promote anti-tumor immunity, shows promising progression-free survival and recovery in many patients. However, acquired resistance is still observed and diagnosed in PD-1 antibody treatments (121, 122). In the Sunbelt Melanoma Trial with a high dose of interferon, the results showed no survival benefit for patients with a single positive sentinel lymph node (stage III) (123). Therefore, alternative strategies of PD-1/PD-L1 inhibition therapy are also required to overcome acquired resistance, provide more options in new therapeutic strategies, and eventually improve clinical achievements for patients with melanoma.

## Author Contributions

XS, NZ, and XL conceived and wrote this manuscript. XL, CY, and BZ discussed the literature and contributed to the revision of the manuscript. All authors have read and approved the final manuscript.

## Conflict of Interest

The authors declare that the research was conducted in the absence of any commercial or financial relationships that could be construed as a potential conflict of interest.
